# Time for action: actinium-225 PSMA-targeted alpha therapy for metastatic prostate cancer - a systematic review and meta-analysis

**DOI:** 10.7150/thno.106574

**Published:** 2025-02-20

**Authors:** Gaia Ninatti, Pietro Scilipoti, Cristiano Pini, Francesco Barletta, Mattia Longoni, Fabrizia Gelardi, Martina Sollini, Giorgio Gandaglia, Mike Sathekge, Francesco Montorsi, Arturo Chiti, Alberto Briganti

**Affiliations:** 1 Nuclear Medicine Department, IRCCS San Raffaele Hospital, Milan, Italy.; 2 School of Medicine and Surgery, University of Milano-Bicocca, Monza, Italy.; 3 Unit of Urology/Division of Oncology, Gianfranco Soldera Prostate Cancer Lab, IRCCS San Raffaele Scientific Institute, Italy.; 4 Faculty of Medicine, Vita-Salute San Raffaele University, Milan, Italy.; 5 Department of Nuclear Medicine, University of Pretoria & Steve Biko Academic Hospital, Pretoria, South Africa.; 6 Nuclear Medicine Research Infrastructure (NuMeRI), Steve Biko Academic Hospital, Pretoria, South Africa.

**Keywords:** PSMA, targeted alpha therapy, prostate cancer, mCRPC, PSA response

## Abstract

**Rationale:** Metastatic prostate cancer in the castration-resistant (mCRPC) setting remains challenging to treat. Prostate-specific membrane antigen (PSMA)-targeted alpha therapy (TAT) is emerging as a promising option. We aimed to systematically review the efficacy and safety of PSMA-TAT in patients with prostate cancer.

**Methods:** A comprehensive search of PubMed/MEDLINE and EMBASE databases was conducted up to October 2024, adhering to the PRISMA guidelines. Selected studies were original research articles evaluating the efficacy and/or safety of PSMA-TAT including at least 10 patients. The outcomes measured included any prostate-specific antigen (PSA) response, ≥50% PSA reduction (PSA50), progression-free survival (PFS), overall survival (OS), and adverse events. PSA50 was pooled using a random-effects model, incorporating individual patient data on PSA50 and previous lines of treatment.

**Results:** Eighteen studies involving 1,155 patients met the inclusion criteria. The majority included heavily pre-treated patients. The most commonly employed radiopharmaceutical was [^225^Ac]Ac-PSMA-617, in 15 studies. The pooled PSA50 response rate was 65% [95% Confidence interval (CI), 57-72%] with a moderate level of heterogeneity (I² = 81.17%, p < 0.001). Pooled response rates in patients who received none, one, and more than one prior line of treatment were 82% (95% CI, 73-90%), 72% (95% CI, 56-85%), and 55% (95% CI, 48-63%), respectively. PFS varied from 3 to 15 months, and OS from 8 to 31 months. Adverse events were predominantly mild (grades 1-2); severe adverse events (≥ grade 3) included anaemia (11%) and thrombocytopenia (6%).

**Conclusion:** PSMA-TAT holds promising efficacy and an acceptable safety profile for treating metastatic prostate cancer. Randomised controlled trials are needed to optimise treatment protocols toward the implementation of PSMA-TAT into clinical practice.

## Introduction

Advanced prostate cancer is associated with a poor prognosis, especially in the metastatic castration-resistant setting (mCRPC) 1. Over the past few years, the treatment landscape for mCRPC has evolved significantly, with therapeutic options now including androgen-axis-pathway inhibitors (ARPIs), taxane-based chemotherapy, and radium dichloride 2. Although these agents have significantly improved survival outcomes in mCRPC, many patients might experience only limited clinical benefits and ultimately face disease progression, prompting the search for new therapeutic strategies.

Prostate-specific membrane antigen (PSMA)-targeted radioligand therapy (RLT) employs radiolabeled molecules that bind to PSMA - a transmembrane glutamate carboxypeptidase highly expressed on prostate cancer cells - to deliver potent radiation doses directly to malignant cells. This strategy has demonstrated high efficacy in numerous studies utilising PSMA-targeted molecules labeled with lutetium-177, a beta-emitting isotope. This has led to the approval of lutetium-based RLT for mCRPC by the European Medicines Agency and the U.S. Food and Drug Administration in 2022. However, despite the significant benefits of [^177^Lu]Lu-PSMA-617 reported by randomised clinical trials 3-5, including survival improvement for patients treated with RLT in addition to standard-of-care compared to standard-of-care alone 6, a substantial proportion of patients does not respond to lutetium-based RLT 7. For these patients, targeted alpha therapy (TAT) - which utilizes alpha-emitting isotopes, most commonly actinium-225 - may offer advantages, potentially enhancing the therapeutic efficacy of PSMA-targeted radioligand therapy 8. Alpha particles, being significantly more energetic than the beta particles emitted by lutetium-177, are able to induce double-stranded DNA damage, killing tumour cells with fewer DNA hits 9. Recent innovative *in-silico* approaches further supported the outperforming properties of alpha particles over beta particles, particularly under hypoxic conditions, in case of low prostate cancer cell density or lower PSMA expression 10,11. Additionally, the shorter range of alpha particles can considerably limit the radiation damage to surrounding healthy organs 9.

Recently, TAT has gained significant attention and several studies have explored its potential in treating various solid tumours, yielding encouraging results and further increasing interest in this therapeutic approach. However, the benefits of TAT in prostate cancer have not been established yet. Indeed, published studies are scattered and heterogeneous, and available literature reviews on TAT only provide a limited overview of the current status of research in the field 12-14. Recently, Dai *et al.* published a more comprehensive, though still partial, meta-analysis article on RLT in metastatic prostate cancer, focusing both on actinium-225 and lutetium-177-based RLT 15.

The present paper aims to provide a comprehensive and up-to-date systematic review of the efficacy and safety of PSMA-TAT in patients with prostate cancer. Furthermore, we aim to produce a meta-analysis on TAT efficacy stratified according to prior lines of systemic treatment and other clinical data, with the overarching goal of highlighting the untapped potential of this therapeutic strategy, informing future large-scale clinical trials towards its adoption in clinical practice.

## Methods

The present systematic review was conducted in accordance with the latest Preferred Reporting Items for Systematic Reviews and Meta-Analyses (PRISMA) guidelines 16 and was registered in the International Prospective Register of Systematic Reviews, PROSPERO (ID: CRD42024529258) (Supplementary Material).

### Search strategy and selection criteria

Original research articles assessing the efficacy and safety of PSMA-TAT in patients with prostate cancer were included. The following exclusion criteria were used: (a) review articles, meta-analyses, guidelines, case reports, case series, editorials, book chapters, and conference abstracts; (b) studies with outcomes available for fewer than ten patients; (c) preclinical studies not involving human subjects; (d) articles not in the English language; (e) studies on PSMA-targeted alpha/beta combined/tandem therapies.

A systematic literature search was performed using the PubMed/MEDLINE and EMBASE databases on April 2^nd^, 2024, and was updated on October 31^st^, 2024. The following search string was used: ("prostate" OR "mCRPC" OR "mCSPC" OR “mHSPC” OR "PC" OR “PCa” OR “Pca”) AND ("PSMA" OR “prostate-specific membrane antigen”) AND ("alpha" OR "α" OR "TAT" or "225Ac" OR "actinium" OR "211At" OR "astatine" OR "227Th" OR "thorium" OR "223Ra" OR "radium" OR "212Pb" OR "lead" OR "212Bi" OR "213Bi" OR "bismuth" OR "149Tb" OR "terbium"). Additionally, the reference lists of included articles were manually screened.

After the removal of duplicates, two authors (M.L. and G.N.) independently performed a preliminary screening of the titles and abstracts of retrieved articles using Rayyan (Rayyan Systems, Cambridge, MA, USA) 17. Any disagreement was resolved by a third reviewer (P.S.) using the majority vote. Finally, the full texts of selected studies were screened for compliance with the eligibility criteria.

### Data analysis

A database was created using Excel® 2023 (Microsoft®, Redmond, WA) for the synthesis of included articles. Two reviewers independently collected the following data: number of patients included, clinical characteristics (baseline PSA value, ISUP group, Eastern Cooperative Oncology Group (ECOG) performance status performance status, previous lines of treatment), presence and site of metastatic disease, details of TAT treatment (radiopharmaceutical, administered activity, number of cycles, intervals between cycles, reasons for treatment discontinuation), survival and efficacy outcomes (progression-free survival, overall survival, PSA response rate), and TAT-related adverse events (fatigue, nausea, anaemia, leukopenia, thrombocytopenia, renal function impairment, xerostomia).

Data about progression-free survival (PFS) and overall survival (OS) were summarised by reporting the median and 95% confidence interval for each study. When data were missing, we noted this explicitly. For analyses of treatment-related adverse events, we pooled patient data from included studies and classified toxicities by severity (all grades vs. grade 3 or higher). If data were missing for some patients, we noted this and only considered those for whom data were available.

The PSA response rate (PSA50) was defined as the percentage of patients achieving a ≥50% reduction in PSA from baseline. When possible, PSA50 was stratified according to previous lines of systemic therapy in the mCRPC setting, according to previous exposure to ARPIs, taxane-based chemotherapy, and [^177^Lu]Lu-PSMA RLT, and according to the presence of visceral metastases. Progression-free survival (PFS) was defined as the time from the initiation of treatment or randomisation (in the case of randomised controlled trials, RCTs) to disease progression or death. OS was defined as the time from the initiation of treatment or randomisation (in the case of RCTs) to death from any cause. TAT-related adverse events were defined according to the Common Terminology Criteria for Adverse Events version 5.

When details on each single patient's clinical data, in particular regarding previous lines of treatment and response to TAT, were not retrievable from the article, we contacted the corresponding author of the manuscript to obtain missing information.

To determine the risk of bias in each selected study, two reviewers (G.N. and P.S.) independently analysed the articles using the Cochrane Collaboration's Risk of Bias (RoB) tool (version 2) for RCTs and the Risk of Bias in Non-randomized Studies - of Interventions (ROBINS-I) tool for non-randomised studies. Any disagreement was resolved by a third author (C.P.).

The total number of patients treated with PSMA-TAT and the total number of patients who achieved the outcome event, i.e. PSA50, and individual treatment data, were extracted from each included study as raw data. Only studies that provided complete outcome data were included in the meta-analysis. The estimated proportion of treatment efficacy was pooled using a random-effects model based on the DerSimonian and Laird method, thereby accounting for both within-study and between-study variability. The Freeman-Tukey double arcsine transformation was employed to stabilise the variance for proportions approaching 0 or 1 18. Forest plots were constructed to illustrate the estimated proportions of patients achieving a ≥50% reduction in PSA levels for each study, alongside their 95% confidence intervals (CI) and the relative weight of each study. The I² statistic and Cochran's Q test were used to evaluate the consistency of the data between studies. The degree of heterogeneity was classified as low (>25%), moderate (>50%), or high (>75%) 19. In the event of high heterogeneity, subgroup analyses were conducted based on previous lines and categories of therapy to identify potential sources of variability. The presence of a moderate level of heterogeneity was considered acceptable. The statistical significance of observed differences between the various groups was determined using the Z-test. All statistical analyses were conducted using the "metaprop" command in STATA (version 16.1; StataCorp LP, College Station, TX, USA).

## Results

### Study selection

**Figure 1:**
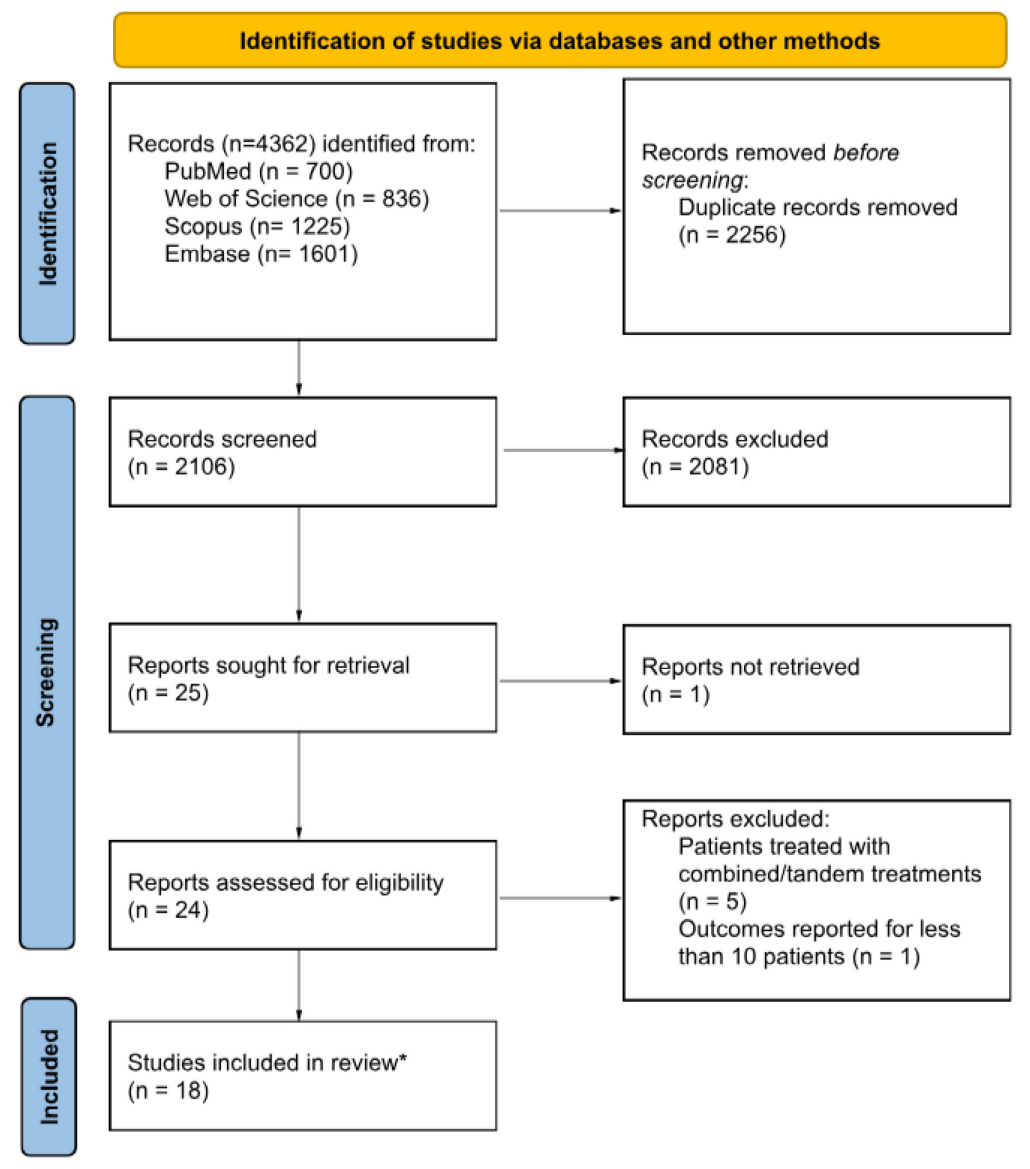
CONSORT flowchart of the study selection process.

Overall, 4,362 references were identified from the systematic literature search. Following the removal of 2,256 duplicates, the titles and abstracts of the remaining records were screened, resulting in the exclusion of an additional 2,081 studies. Subsequently, the full texts of 24 articles were assessed, leading to the exclusion of five studies on patients treated with combined/tandem PSMA-targeted alpha/beta therapy and one study reporting outcome data for less than 10 patients. Ultimately, 18 original research studies meeting the inclusion criteria were incorporated into this systematic review. The study selection process is illustrated in **Figure 1**.

### Study characteristics

The 18 selected studies collectively included data from 1,155 patients 20-37.

Except for one multicentre retrospective cohort study 20, one dual-centre phase I dose escalation trial 21, and one single-centre prospective study 22, all others were single-centre retrospective studies.

Overall, the median patient age was 69 years (range 37-90), and the median ECOG performance status was 1 (range 0-4).

Most individuals had mCRPC (1,134 out of 1,155, 98%), while a minority (25 out of 1,155, 2%) had metastatic hormone-sensitive prostate cancer (mHSPC).

The median PSA value at baseline was 169 ng/mL (range 0.349-7,168 ng/mL).

Data on bone metastases were available for all patients, while information on lymph node and visceral metastases were not available for 134 and 159 patients, respectively. Most patients had metastatic disease in the bones (1,037 out of 1,155, 93%) and lymph nodes (734 out of 1,021, 72%), whereas a smaller proportion had visceral metastases (215 out of 996, 22%).

Most studies included heavily pretreated patients, except for one that included only treatment-naïve mHSPC patients (21/21, 100%) 23, one that included 4/17 (24%) treatment-naïve and 13/17 (76%) chemotherapy and ARPi-naïve patients 24, and one that included only mCRPC patients in the post androgen deprivation therapy setting (53/53, 100%) 25.

Clinical characteristics of the included patients are summarised in **Table 1.**

### Risk of bias and heterogeneity

All 18 studies exhibited a moderate risk of bias, primarily due to their retrospective design; in six studies, there was a moderate risk of bias due to missing data. **Supplementary Figure 1** delineates the assessments for each domain across all included studies.

### Radiopharmaceuticals and treatment protocols

All included studies utilised the alpha emitter actinium-225 for PSMA-TAT. The specific radiopharmaceutical was documented for 667 out of 1,155 patients: most patients were treated with [^225^Ac]Ac-PSMA-617 (621 out of 667, 93%) 16-21,23-32, while a smaller proportion received [^225^Ac]Ac-PSMA-I&T (14 out of 667, 2%) 37 and [^225^Ac]Ac-J591 (32 out of 667, 5%) 21.

Treatment regimens varied in terms of administered activity, time between cycles, and number of cycles (see Table 1).

In 8 out of 18 studies, the administered activity was calculated by patient's weight, specifically 100 kBq/kg in 7 studies 22,26,27,31,32,37 and 100-150 kBq/kg in one study 29. In 8 out of 18 studies, all patients received an initial activity of 8 MBq, followed by de-escalation based on the response to each earlier administered treatment cycle and/or for salivary gland protection 20,23-25,30,35. In one of the remaining studies, authors conducted a phase I dose-escalation trial from 13.3 to 93.3 kBq/kg 21. Finally, in the other remaining study, the criteria for determining the administered activity were not specified 33.

Sixteen studies performed cycles of PSMA-TAT every 8 weeks, with one study allowing for an interval of 8-12 weeks 34, and another of 8-28 weeks 27.

Overall, the median number of treatment cycles was 2.5 (range 1-9).

### Efficacy of PSMA-TAT

All studies reported outcomes in terms of biochemical response (PSA50) and 14 out of 18 also reported survival outcomes (PFS and/or OS).

The median follow-up time was reported in 10 studies, ranging from 5.4 to 22 months, with a median of 9 months.

Overall, the median PSA50 response across studies was 65%, with a range from 26 to 91%. Particularly, the median PSA50 response was 68% in 617 patients treated with [^225^Ac]Ac-PSMA-617, 50% in 14 patients treated with [^225^Ac]Ac-PSMA-I&T, and 47% in 32 patients treated with [^225^Ac]Ac-J591. PSA50 response data related the radiopharmaceutical used were not available for 492 patients.

The studies reporting the lowest PSA50 included a cohort of heavily pretreated patients who all experienced disease progression under previous treatment with [^177^Lu]Lu-PSMA-617 (PSA50: 26%) 27, and a cohort of patients with a median ECOG PS of 3 before treatment, where a large majority of them (86%) had received three or more previous lines of therapy for mCRPC (PSA50: 39 %) 22.

Conversely, the three studies demonstrating a higher PSA50 featured a cohort of mCRPC patients in the post-androgen deprivation setting (PSA50: 91%) 25, a mixed cohort of patients partly in the post-androgen deprivation setting and partly with mHSPC (PSA50: 88%) 24, and a cohort of treatment-naïve mHSPC patients (PSA50: 86%) 23, respectively.

**Table 2** shows PSA50 response rates stratified according to previous lines of treatment for mCRPC.

**Supplementary Table 1** shows PSA50 response rates for the two most common [^225^Ac]Ac-PSMA-617 RLT treatment regimens (i.e. 8 MBq followed by de-escalation every 8 weeks and 100 kBq/kg every 8 weeks).

The median PFS and OS times were reported in 12 studies. The median PFS ranged from 3 to 15 months, while the median OS varied from 8 to 31 months. The three studies with the lowest reported median PFS and OS - specifically, PFS times of 3, 3.5, and 4 months and OS times of 8, 8, and 10 months 26,27,33 - uniformly included cohorts of heavily pretreated patients. In contrast, the majority of the four studies that documented the highest median PFS and OS times - PFS of 12, 14, 15 months and OS of 17, 15, 18, and 31 months 22,23,28,36 - enrolled patients who were either treatment-naïve or had previously received only androgen deprivation therapy or one line of therapy, except for one study that included heavily pretreated patients 22.

### Meta-analysis

**Figure 2:**
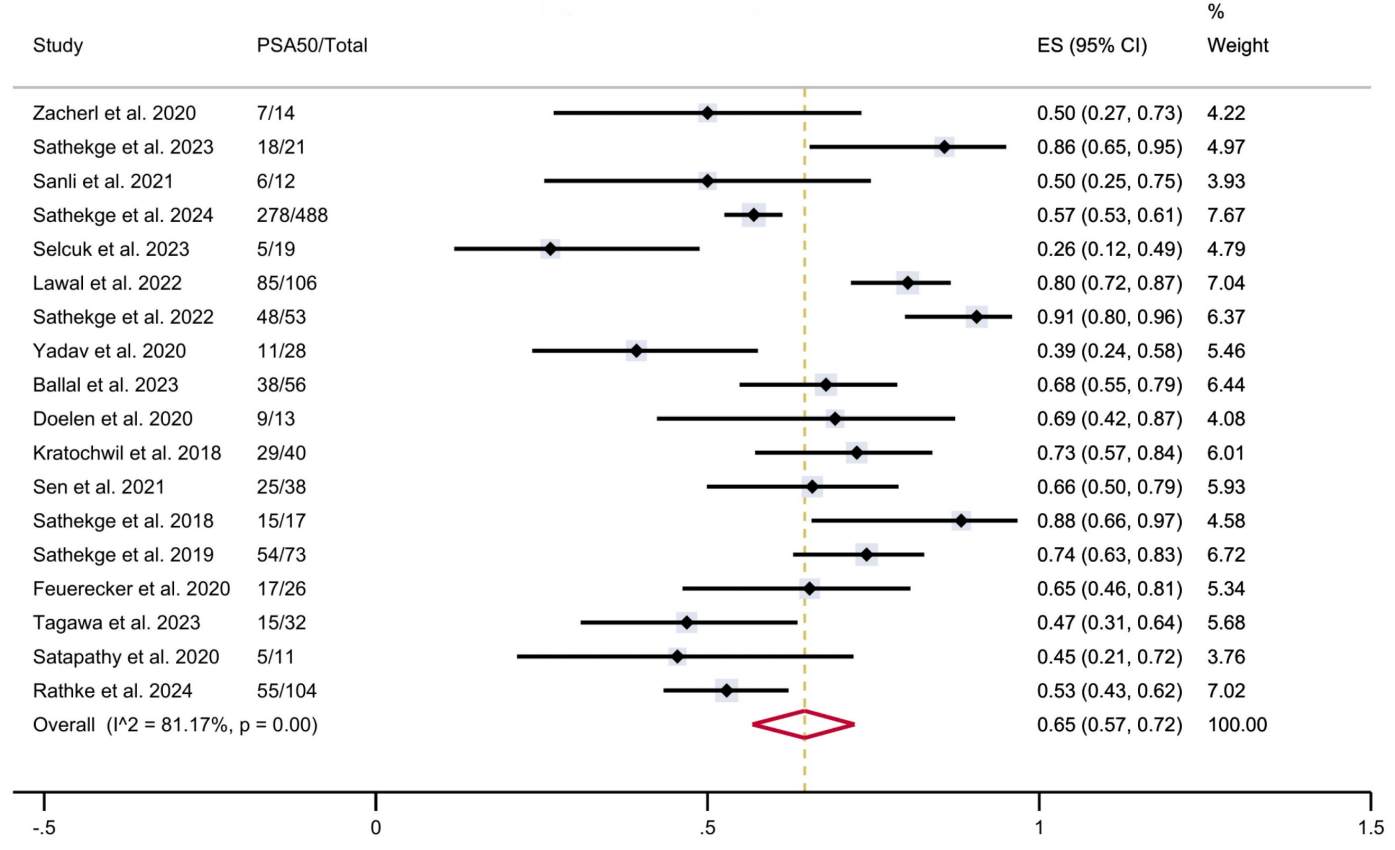
PSA50 response rates in included studies. CI: confidence interval; ES: effect size; PSA50: ≥50% decline in PSA value from baseline.

A total of 18 studies were considered eligible for the meta-analysis, collectively including 1,151 patients who had been treated with PSMA-TAT with available data on PSA response. The estimated pooled proportions of patients achieving a ≥50% reduction in PSA levels following PSMA-TAT in the overall population was 65% (95% CI 57-72%), with high between-study statistical heterogeneity (I²=81.17%, p<0.001) (**Figure 2**).

**Figure 3:**
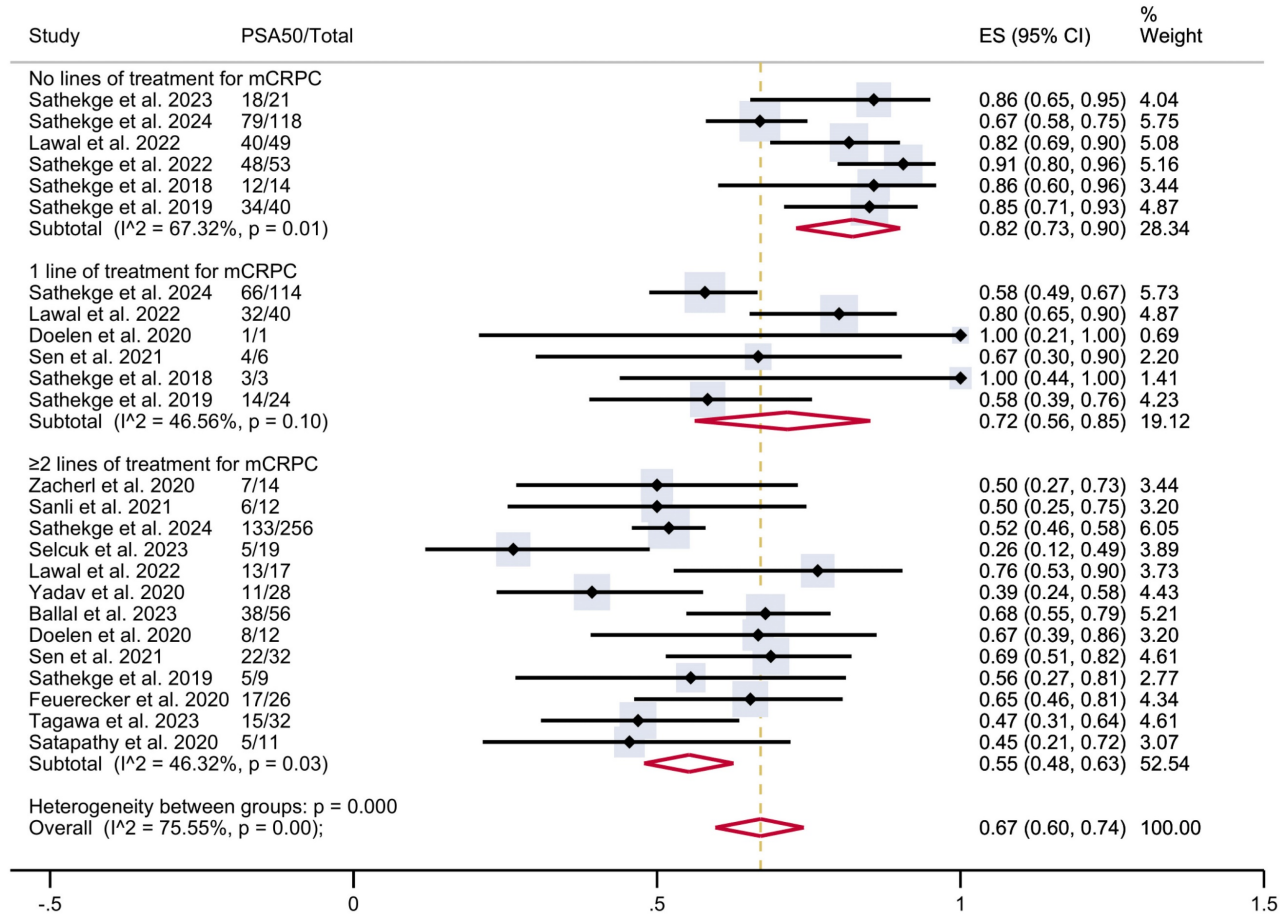
PSA50 response rates in included studies with patients stratified according to the previous lines of therapy for mCRPC. CI: confidence interval; ES: effect size; mCRPC: metastatic castration-resistant prostate cancer; PSA50: ≥50% decline in PSA value from baseline.

To investigate this heterogeneity, a subgroup analysis was conducted according to the number of prior lines of treatment received before PSMA-TAT, when that information was available (**Figure 3**). The analysis included six studies with patients who had not received any prior lines of treatment for mCRPC (n = 295), six studies with patients who had received one prior line of treatment (n = 188), and 13 studies with patients who had undergone two or more prior lines of treatment (n = 524). In patients with no previous treatment, the estimated PSA50 response rate was 82% (95% CI 73-90%). In patients who had undergone one prior line of treatment, the proportion was 72% (95% CI 56-85%), while in those who had undergone two or more prior lines of treatment, it was 55% (95% CI 48-63%). These results were associated with moderate heterogeneity within each group. The overall pooled estimate PSA50 response rate for all studies was 67% (95% CI 60-74%), associated with a moderate overall heterogeneity (I² = 75.55%, p < 0.001).

Further subgroup analyses were conducted based on the category of prior treatment received before PSMA-TAT, including ARPI, taxane-based chemotherapy, and radioligand therapy, as well as the presence of visceral metastases.

**Figure 4:**
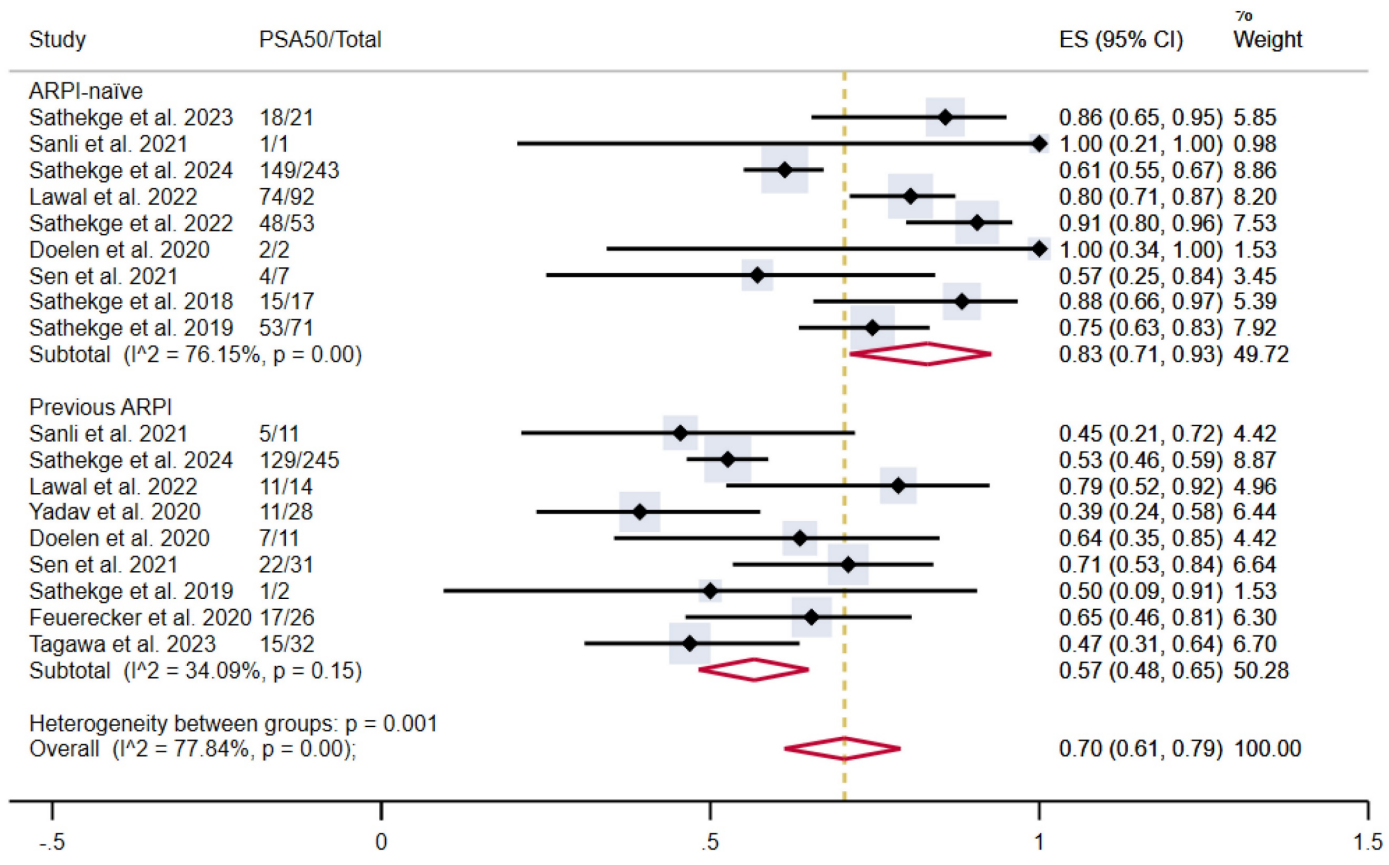
PSA50 response rates in included studies with patients stratified according to previous treatment with ARPI. ARPI: androgen receptor pathway inhibitor; CI: confidence interval; ES: effect size; PSA50: ≥50% decline in PSA value from baseline.

The pooled estimated proportion of patients achieving PSA50 in patients not previously treated with ARPI was 83% (95% CI 71-93%), compared to 57% in patients who had previously undergone ARPI (95% CI 48-65%), with moderate and low heterogeneity, respectively. The overall pooled proportion was 70% (95% CI 61-79%) (**Figure 4**).

Patient outcomes are summarised in **Table 1.**

**Figure 5:**
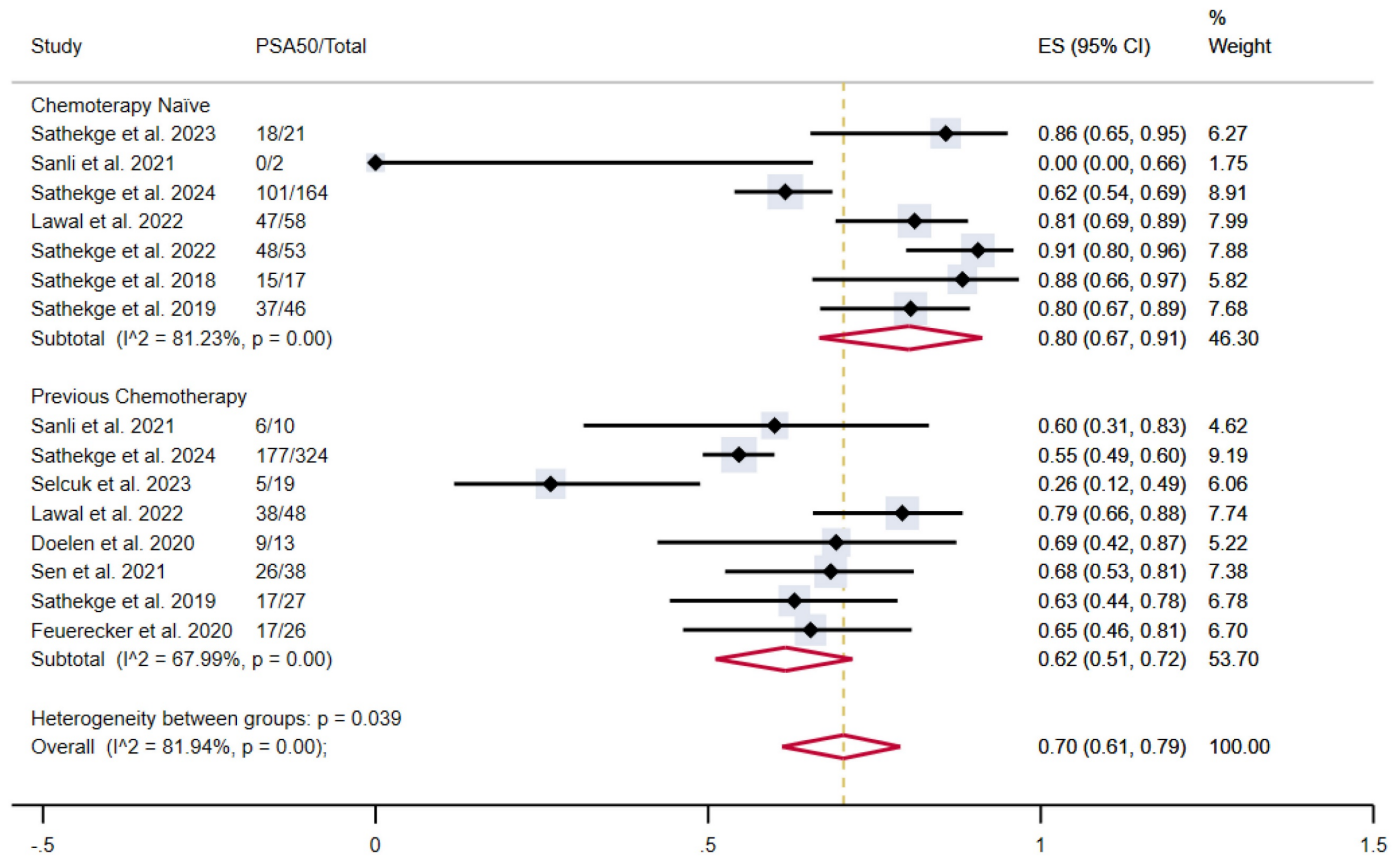
PSA50 response rates in included studies with patients stratified according to previous taxane-based chemotherapy. CI: confidence interval; ES: effect size; PSA50: ≥50% decline in PSA value from baseline.

Similarly, chemotherapy-naïve patients showed a proportion of outcome achievement of 80% (95% CI 67-91%) versus 62% (95% CI 51-72%) in patients who underwent previous taxane-based chemotherapy, with moderate and high heterogeneity, respectively. The overall pooled proportion was 70% (95% CI 61-79%) (**Figure 5**).

**Figure 6:**
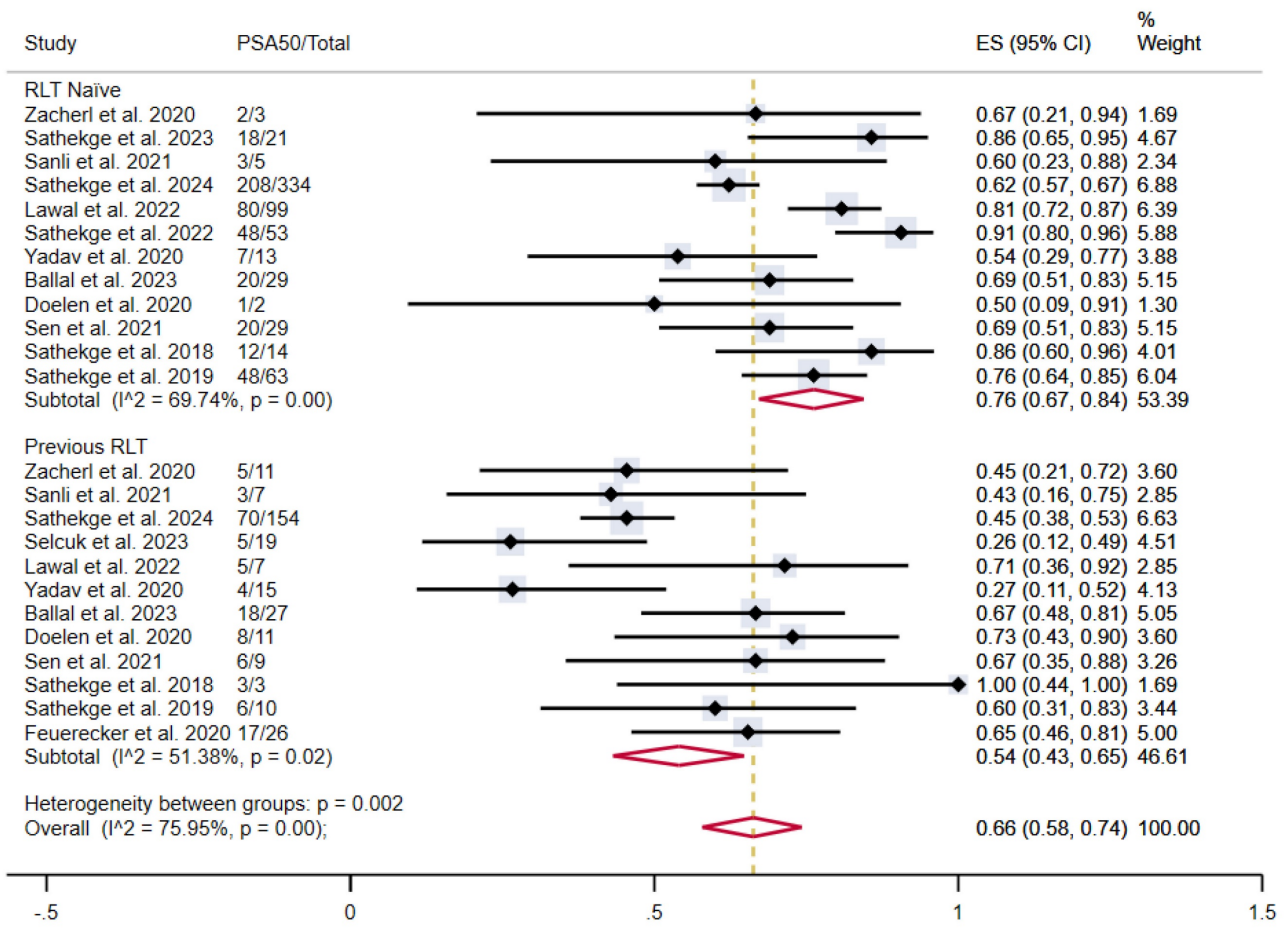
PSA50 response rates in included studies with patients stratified according to previous lutetium-177-based RLT. CI: confidence interval; ES: effect size; PSA50: ≥50% decline in PSA value from baseline; RLT: radioligand therapy.

In patients who were naïve to RLT, the proportion of patients achieving PSA50 was 76% (95% CI 67-84%), versus 54% (95% CI 43-65%) in patients previously treated with [^177^Lu]Lu-PSMA-617, both with moderate heterogeneity (**Figure 6**).

**Figure 7:**
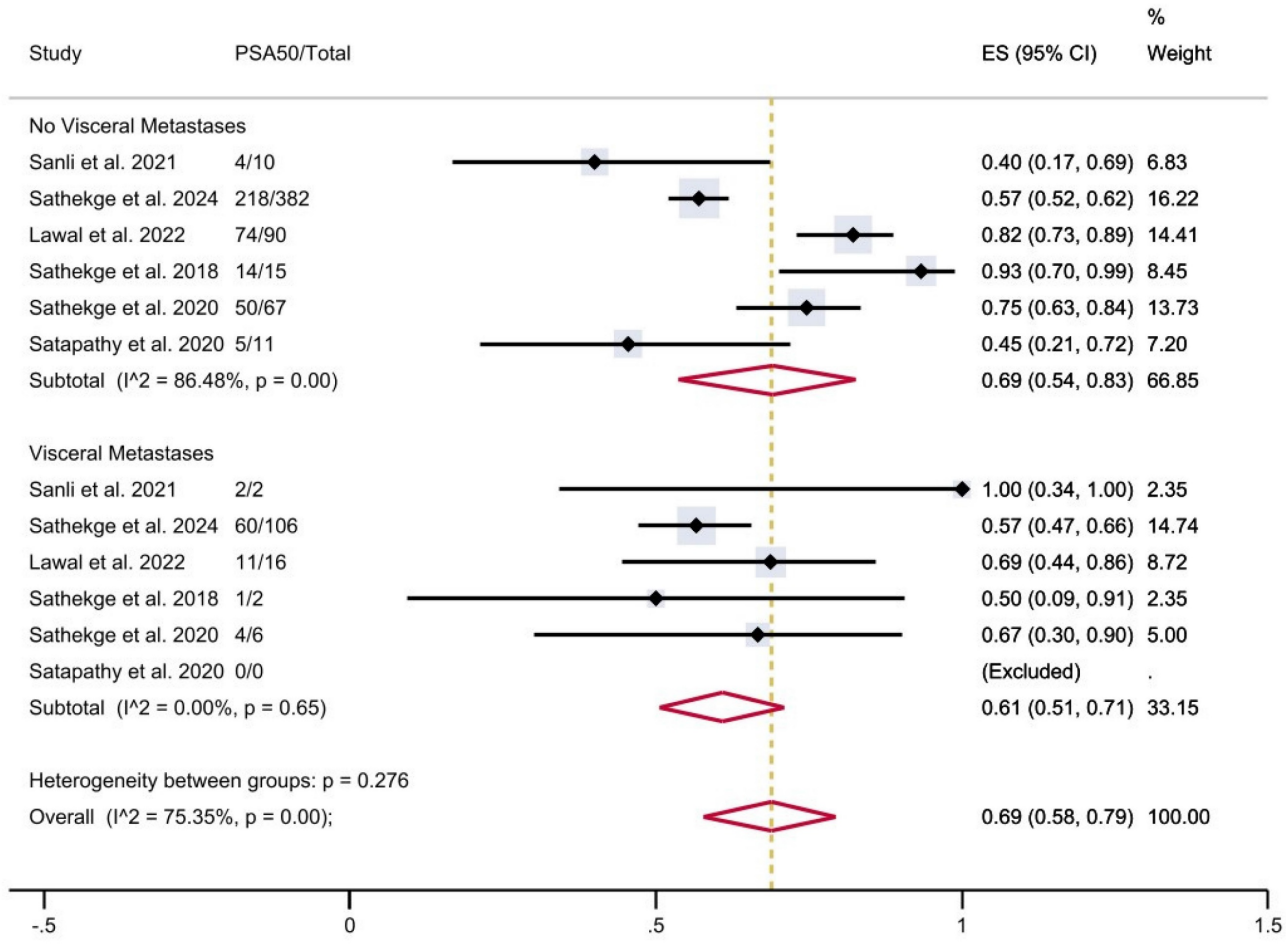
PSA50 response rates in included studies with patients stratified according to the presence of visceral metastases. CI: confidence interval; ES: effect size; PSA50: ≥50% decline in PSA value from baseline.

Finally, in patients with visceral metastases, the proportion of patients achieving PSA50 was 61% (95% CI 51-71%), versus 69% (95% CI 54-83%) in patients without visceral metastases (**Figure 7**), with a moderate overall heterogeneity.

A significant difference in treatment efficacy was observed across the subgroups in all the aforementioned analyses (p < 0.001).

Subanalyses based on the type of radiopharmaceutical used and treatment regimen were not performed due to a partial lack of data and to significant imbalance between groups.

### Adverse events of PSMA-TAT

Adverse events were only partially documented in the studies included.

Many studies did not report the rate of treatment discontinuation due to adverse events. Among those that did, toxicity-related suspension rates varied significantly, with one study noting a rate of 3.6% 29 and another of 31% 33.

The majority of reported adverse events were either mild or moderate (grade 1 and 2), accounting for 89% of all reported side effects. The most common severe adverse events (grade ≥3) were anaemia (11%) and thrombocytopenia (6%).

**Table 3** summarises the adverse events by type and severity.

## Discussion

This systematic review highlighted the potential of actinium-based PSMA-TAT in the treatment of advanced prostate cancer. The estimated pooled proportion of patients achieving a ≥50% reduction in PSA levels following PSMA-TAT in the overall population - a proxy of treatment efficacy - was 65%. This datum, higher than what reported for lutetium-based RLT (49%, 15) is consistent with other meta-analyses (65% vs 59-63%, 11-13, 38, although our analysis included more patients (1,155), without overlaps among series and populations, both retrospective and prospective studies, as well as trials with PSMA molecules other than PSMA-617, though this radiopharmaceutical was the most extensively explored. Notably, although the median PSA50 response rate was higher for patients treated with [225Ac]Ac-PSMA-617 than for both [225Ac]Ac-PSMA-I&T and [225Ac]Ac-J591, conclusions about differences in PSMA ligand efficacy (PSMA-617 vs. PSMA-I&T vs. J591) should be drawn with caution, as disparities in numbers and populations are likely responsible for the variability of PSA50 responses.

The already encouraging result on PSMA-TAT efficacy becomes even more interesting when considering our stratification by the number and types of prior systemic treatments.

Indeed, while it is mandatory to underscore that data from this systematic review, which includes mostly retrospective studies, and randomised controlled trials are not directly comparable, collating the efficacy of PSMA-TAT to other available treatments approved in clinical practice can provide valuable context. For instance, patients from all included studies who received PSMA-TAT in the first-line therapeutic setting for mCRPC showed a pooled estimate PSA50 of 82%. This figure stands out when considering the performance of docetaxel in the same setting in the TAX327 study, which evaluated a cohort comprised of only 12% of patients with ECOG ≥2 and obtained a PSA50 of 48% 39. First-line therapy with enzalutamide in mCRPC demonstrated similar PSA50 rates, with 78% and 82% in the PREVAIL and TERRAIN trials, respectively 40,41.

Similarly, patients included in this systematic review who received PSMA-TAT for mCRPC after taxane-based chemotherapy demonstrated a PSA50 of 62%. This compares favourably with available data for cabazitaxel (TROPIC) 42, abiraterone acetate (COU-AA-301) 43, and enzalutamide (AFFIRM) 44, with respective PSA50 rates of 39%, 29%, and 54%. Lutetium-177-based radioligand therapy in a similar setting, as evaluated by the TheraP trial, showed a PSA50 of 66% 45.

Results remain encouraging when considering patients in the mCRPC setting after two or more lines of therapy, where a PSA50 of 54% for PSMA-TAT compares with cabazitaxel in the CARD trial and [^177^Lu]Lu-PSMA-617 in the VISION trial 4, which showed a PSA50 of 36% and 46%, respectively.

More broadly, it is notable that higher PSA50 response rates were observed in studies involving patients in earlier disease stages. This is evident when stratifying patients according to previous lines of therapy for mCPRC, and according to previous treatments with ARPIs, taxane-based chemotherapy, and lutetium-177-based RLT.

Unfortunately, the lack of homogeneous information regarding follow-up and PFS assessment prevents us from conducting a robust meta-analysis on PFS and OS. Data on PSA50 response are definitely not enough to determine practice changes, but can serve as a proxy of treatment efficacy. Dai *et al.* 15, who meta-regressed data from three studies, demonstrated that patients treated with TAT exhibiting PSA responses had significantly improved PFS and OS, underscoring the correlation between survival and biochemical efficacy outcomes.

Overall, these data further highlight the untapped potential of PSMA-TAT and warrant investigations on this therapeutic option as early as possible in the natural history of metastatic prostate cancer.

The safety profile of PSMA-TAT was generally favourable, with most adverse events being mild to moderate. The most frequent adverse event, occurring in 77% of patients, was xerostomia, which is attributable to the high expression of PSMA in salivary glands. However, severe cases (grade 3 or higher) were rare, occurring in only 2% of treated patients. Moreover, although there is still no consensus on how to mitigate salivary gland toxicity in PSMA-targeted radioligand therapy, many strategies are under investigation, including external salivary gland cooling, intravenous hydration, botulinum toxin injection, and administration of oral monosodium glutamate or folic polyglutamate 46. Overall, severe (grade ≥3) adverse events, more frequently anemia (11%) and thrombocytopenia (6%), were relatively uncommon. Additionally, the pooled account of hematologic adverse events may be overestimated, as many patients were heavily pretreated and already presented some degree of hematologic impairment before PSMA-TAT.

A major limitation identified in this review is the heterogeneity of the study populations and treatment protocols. Additionally, most studies were retrospective, which introduces biases and limits the ability to draw definitive conclusions. The differences in administered activities, cycle numbers and intervals, and baseline patient characteristics (most notably the number of prior treatment lines, prevalence of visceral metastases, and functional status) complicate direct comparisons and synthesis of data. It would be of interest to further stratify patients based on additional factors and parameters, such as performance status, number of metastases, tumour burden, and blood test results, which were unfortunately unavailable in the majority of cases. These indices could provide information on general patients' condition and therefore highlight potential study biases. Remarkably, our analyses demonstrate that TAT performed better in patients without visceral metastases; however, this finding could be influenced by many confounding factors.

Overall, the lack of randomised controlled trials means that most findings are based on observational data, which can all be influenced by confounding factors. Nonetheless, although preliminary and burdened by some limitations, our results outlined the high potential of TAT: this treatment shows an efficacy comparable with the one obtained in clinical trials with other now-approved drugs, with a favourable safety profile.

There are currently more than ten phase I/II ongoing clinical trials evaluating PSMA-TAT as a single agent in prostate cancer in different settings and with various radiopharmaceuticals (https://clinicaltrials.gov, https://euclinicaltrials.eu). Several next-generation optimised PSMA-targeting molecules, with a more favourable biodistribution profile, are under investigation with promising preclinical results, such as [^225^Ac]Ac-FL-020, [^225^Ac]Ac-PSMA-R2, [^225^Ac]Ac-PSMA-Trillium, [^225^Ac]Ac-macropa-pelgifatamab, and [^225^Ac]Ac-PSMA-62. Although still mostly characterised by heterogeneous and fragmented approaches, it is to be expected that these studies will further consolidate data on the efficacy of PSMA-TAT and establish the foundations for future phase III trials. Moreover, other alpha emitters such as lead-212 and astatine-211 are gaining attention.

Overall, the increasing availability of both alpha and beta emitters for PSMA-targeted therapy raises numerous questions that future studies will need to address, particularly concerning the advantages of using alpha versus beta emitters depending on the clinical setting and the specific indications for each. Strategies combining beta and alpha PSMA-targeted therapy (i.e., cocktail therapy), as well as sequential use of alpha and beta emitters (i.e., tandem therapy) will need to be further explored, as many patients progressing to beta respond to alpha emitters. Additionally, clinical trials are needed to assess the potential synergistic effects of PSMA-TAT in combination with other agents, such as ARPIs, immunotherapies, PARP inhibitors, and taxanes.

Overall, this systematic review underscores the great potential of PSMA-TAT in metastatic prostate cancer, especially in the earlier disease stages. The significant cytotoxic effect of alpha particles can overcome resistances and exert therapeutic effects even in challenging scenarios, all while maintaining a favourable safety profile. Moreover, the possibility of selecting patients for treatment and monitoring response by *in vivo* PET imaging of the same molecular target, PSMA, offers this therapy the unique advantage of theranostics.

The overarching goal of this work is not only to provide clinicians with updated evidence on the efficacy and safety of PSMA-TAT but also to underscore its potential to drive the design of prospective, randomised controlled trials and facilitate the introduction of this therapy into clinical practice, to the ultimate benefit of prostate cancer patients. The promising results highlighted by this systematic review should encourage further investigations to optimise treatment protocols, identify the ideal patient population, and explore combination strategies.

In conclusion, PSMA-TAT shows promising efficacy and an acceptable safety profile in treating metastatic prostate cancer. A significant PSA response was reported in a substantial proportion of patients, from heavily pretreated cohorts to earlier disease settings. Adverse events are generally mild and manageable. Data collected and synthesised in this systematic review urge a call for action: this treatment can exert impressive therapeutic effects in this challenging scenario. It is time to confirm these findings and optimise treatment protocols in randomised controlled trials, toward the prompt implementation of PSMA-TAT into clinical practice.

## Supplementary Material

Supplementary figure, table, and information.

## Figures and Tables

**Table 1:** Summary of baseline characteristics and outcomes of studies included in the systematic review and meta-analysis.

Ref	Study design	Patients (n)	Age (mean /median)	ECOG PS (median)	Baseline PSA (median, ng/mL)	Metastases	Prior systemic treatments	Radiopharmaceutical and treatment regimen	Number of cycles (median)	Follow-up time (median)	Criteria to identify progressive disease	Main results
Zacherl *et al.* (2020)	Retrospective	14	75 (median)	1	112	Skeletal: 93% Lymph node: 71% Visceral: 21%	ADT: 100% ARPi: 100% Taxane-based CT: 86% [^177^Lu]Lu-PSMA-617: 79% Radium-223 dichloride: 14%	[^225^Ac]Ac-PSMA-I&T 100 kBq/kg every 8 weeks	2	5.4 months	PSA, PSMA PET/CT	PSA50: 50% Any PSA reduction: 79% mPFS: NA mOS: NA
Sathekge *et al.* (2023)	Retrospective	21	67 (median)	1	197	Skeletal: 100% Lymph node: NA Visceral: 29%	None	[^225^Ac]Ac-PSMA-617 8 MBq followed by de-escalation every 8 weeks	3	NA	PSA, PSMA PET/CT	PSA50: 86% Any PSA reduction: 95% mPFS: NA mOS: 31 months (CI 13-49)
Sanli *et al.* (2021)	Retrospective	12	70 (median)	2	129	Skeletal: 100% Lymph node: 75% Visceral: 17%	ADT: 100% ARPi: 92% Taxane-based CT: 83% [^177^Lu]Lu-PSMA-617: 58% Radium-223 dichloride: NA	[^225^Ac]Ac-PSMA-617 100 kBq/kg every 8 weeks	2	10 months	PSA, PSMA PET/CT	PSA50: 50% Any PSA reduction: 75% mPFS: 4 months (CI NA) mOS: 10 months (CI NA)
Sathekge *et al.* (2024)	Retrospective	488	68 (mean)	1	170	Skeletal: 89% Lymph node: 72% Visceral: 20%	ADT: 86% ARPi: 50% Taxane-based CT: 67% [^177^Lu]Lu-PSMA-617: 32% Radium-223 dichloride: 4%	Radiopharmaceutical NA 8 MBq followed by de-escalation every 8 weeks	2	9 months	Clinical, PSA, imaging	PSA50: 57% Any PSA reduction: 73% mPFS: 8 months (CI 7-9) mOS: 15.5 months (CI 13-18)
Selcuk *et al.* (2023)	Retrospective	23	70 (mean)	NA	104	Skeletal: 91% Lymph node: 56% Visceral: NA	ADT: 100% ARPi: 83% Taxane-based CT: 96% [^177^Lu]Lu-PSMA-617: 100% Radium-223 dichloride: NA	[^225^Ac]Ac-PSMA-617 100 kBq/kg with a median interval of 13 weeks	2	NA	PSA, PSMA PET/CT	PSA50 (after the 1st cycle): 26%* Any PSA reduction (after the 1st cycle): 58%* mPFS: 3 months (CI NA) mOS: 8 months (CI NA)
Lawal *et al.* (2022)	Retrospective	106	69 (mean)	NA	250	Skeletal: 100% Lymph node: 60% Visceral: 15%	ADT: 100% ARPi: 13% Taxane-based CT: 45% [^177^Lu]Lu-PSMA-617: 7% Radium-223 dichloride: 2%	[^225^Ac]Ac-PSMA-617 8 MBq followed by de-escalation every 8 weeks	4	8 months	PSA	PSA50: 80% Any PSA reduction: NA mPFS: 14 months (CI 8-20) mOS: 15 months (CI 13-17)
Sathekge *et al.* (2022)	Retrospective	53	63 (median)	1	466	Skeletal: 89% Lymph node: 68% Visceral: 11%	ADT: 100% ARPi: 0% Taxane-based CT: 0% [^177^Lu]Lu-PSMA-617: 0% Radium-223 dichloride: 0%	[^225^Ac]Ac-PSMA-617 8 MBq followed by de-escalation every 8 weeks	3	NA	PSA, PSMA PET/CT	PSA50: 91% Any PSA reduction: 96% mPFS: NA mOS: NA
Yadav *et al.* (2020)	Prospective	28	70 (mean)	3	222	Skeletal: 96% Lymph node: 86% Visceral: 32%	ADT: 100% ARPi: 100% Taxane-based CT: 93% [^177^Lu]Lu-PSMA-617: 54% Radium-223 dichloride: NA	[^225^Ac]Ac-PSMA-617 100 kBq/kg every 8 weeks	3	10 months	PSA, PSMA PET/CT	PSA50: 39% Any PSA reduction: 89% mPFS: 12 months (CI 9-13) mOS: 17 months (CI 16-NR)
Ballal *et al.* (2023)	Retrospective	56	68 (median)	3	NA	Skeletal: 95% Lymph node: 95% Visceral: 43%	ADT: 100% ARPi: 98% Taxane-based CT: 89% [^177^Lu]Lu-PSMA-617: 48% Radium-223 dichloride: NA	[^225^Ac]Ac-PSMA-617 100-150 kBq/kg every 8 weeks	4	22 months	Imaging	PSA50: 68% Any PSA reduction: 91% mPFS: 9 months (CI 7-15) mOS: 15 months (CI 10-19)
Doelen *et al.* (2020)	Retrospective	13	71 (median)	NA	878	Skeletal: 100% Lymph node: 85% Visceral: 62%	ADT: 100% ARPi: 85% Taxane-based CT: 100% [^177^Lu]Lu-PSMA-617: 15% Radium-223 dichloride: 31%	[^225^Ac]Ac-PSMA-617 8 MBq followed by de-escalation every 8 weeks	3	NA	Clinical	PSA50: 69% Any PSA reduction: 85% mPFS: 5.5 months (CI NA) mOS: 8.5 months (CI NA)
Kratochwil *et al.* (2018)	Retrospective	40	70 (median)	1	169	Skeletal: 98% Lymph node: NA Visceral: 40%	ADT: 100% ARPi: NA% Taxane-based CT: NA% [^177^Lu]Lu-PSMA-617: NA Radium-223 dichloride: 23%	[^225^Ac]Ac-PSMA-617 100 kBq/kg every 8 weeks	3	NA	PSA, PSMA PET/CT	PSA50: 73% Any PSA reduction: 93% mPFS: 7 months (CI NA) mOS: NA
Sen *et al.* (2021)	Retrospective	38	68 (median)	NA	147	Skeletal: 100% Lymph node: 53% Visceral: 18%	ADT: 100% ARPi: 84% Taxane-based CT: 100% [^177^Lu]Lu-PSMA-617: 24% Radium-223 dichloride: 5%	[^225^Ac]Ac-PSMA-617 100 kBq/kg every 8 weeks	2	14 months	PSA, PSMA PET/CT	PSA50: 66% Any PSA reduction: 87% mPFS: 8 months (CI 5-10.5) mOS: 12 months (CI 9-15)
Sathekge *et al.* (2018)	Retrospective	17	65 (mean)	0	49	Skeletal: 82% Lymph node: 53% Visceral: 12%	ADT: 65% ARPi: 0% Taxane-based CT: 0% [^177^Lu]Lu-PSMA-617: 18% Radium-223 dichloride: 0%	[^225^Ac]Ac-PSMA-617 8 MBq followed by de-escalation every 8 weeks	3	13 months	PSA, PSMA PET/CT	PSA50: 88% Any PSA reduction: 94% mPFS: NA mOS: NA
Sathekge *et al.* (2019)	Retrospective	73	69 (median)	0	57	Skeletal: 90% Lymph node: 58% Visceral: 8%	ADT: 100% ARPi: 1% Taxane-based CT: 37% [^177^Lu]Lu-PSMA-617: 14% Radium-223 dichloride: 1%	[^225^Ac]Ac-PSMA-617 8 MBq followed by de-escalation every 8 weeks	3	9 months	PSA	PSA50: 74% Any PSA reduction: 82% mPFS: 15 months (CI 13-17.5) mOS: 18 months (CI 16-20)
Feuerecker *et al.* (2020)	Retrospective	26	73 (median)	1	331	Skeletal: 100% Lymph node: 88% Visceral: 42%	ADT: 100% ARPi: 100% Taxane-based CT: 100% [^177^Lu]Lu-PSMA-617: 100% Radium-223 dichloride: 23%	[^225^Ac]Ac-PSMA-617 Activity and interval NA	2	7 months	Clinical-PSMA PET/CT, PSA	PSA50: 65% Any PSA reduction: 88% mPFS: 3.5 months (CI 2-11) mOS: 8 months (CI 4.5-12)
Tagawa *et al.* (2023)	Phase I open-label dose escalation trial	32	70 (median)	1	149	Skeletal: 97% Lymph node: 88% Visceral: NA	ADT: 100% ARPi: 100% Taxane-based CT: 63% [^177^Lu]Lu-PSMA-617: 47% Radium-223 dichloride: 28%	[^225^Ac]Ac-J591 Single dose, with activity range 13.3-93.3 kBq/kg	1	NA	PSA	PSA50: 47% Any PSA reduction: 72% mPFS: 5.5 months (CI 4-8) mOS: 11 months (CI 6.5-17)
Satapathy *et al.* (2020)	Retrospective	11	68 (median)	1	158	Skeletal: 100% Lymph node: 82% Visceral: 0%	ADT: 100% ARPi: NA Taxane-based CT: NA [^177^Lu]Lu-PSMA-617: 46% Radium-223 dichloride: 0%	[^225^Ac]Ac-PSMA-617 100 kBq/kg every 8-12 weeks	2	NA	PSA	PSA50: 45% Any PSA reduction: 73% mPFS: NA mOS: NA
Rathke *et al.* (2024)	Retrospective	104	62 (median)	1	312	Skeletal: 96% Lymph node: 70% Visceral: NA	ADT: 100% ARPi: 89% Taxane-based CT: 70% [^177^Lu]Lu-PSMA-617: 37% Radium-223 dichloride: NA	[^225^Ac]Ac-PSMA-617 6-8 MBq followed by de-escalation every 8 weeks	2	NA	PSMA PET/CT or SPECT/CT, PSA	PSA50: 53% Any PSA reduction: NA mPFS: NA mOS: 9 months (CI 7-11)

Abbreviations: ADT: androgen deprivation therapy; ARPi: androgen-axis-pathway inhibitors; CI: 95% confidence interval; CT: chemotherapy; ECOG PS: Eastern Cooperative Oncology Group performance status; ISUP: International Society of Urological Pathology; mOS: median overall survival; mPFS: median progression-free survival; NA: not available; NR: not reached; PET: positron emission tomography; PSA: prostate specific antigen; PSA50: ≥50% decline in PSA value from baseline; SPECT: single photon emission computed tomography *data on PSA50 and any PSA reduction available for 19/23 patients.

**Table 2:** PSA50 response rates according to previous therapies for mCRPC.

Variable	Total	PSA50	No PSA 50	*p* value
	n	n (%)	n (%)	
Previous lines of therapy for mCRPC (n=1007)				<0.0001
0	295	231 (78%)	64 (22%)	
1	188	120 (64%)	68 (36%)	
≥2	524	285 (54%)	239 (46%)	
Previous ARPi (n=907)				<0.0001
Yes	400	218 (54.5%)	182 (45.5%)	
No	507	364 (72%)	143 (28%)	
Previous taxane-based CT (n=866)				<0.0001
Yes	505	295 (58%)	210 (42%)	
No	361	266 (74%)	95 (26%)	
Previous [^177^Lu]Lu-PSMA-617 RLT (n=964)				<0.0001
Yes	299	150 (50%)	149 (50%)	
No	665	467 (70%)	198 (30%)	

**Table 3:** Adverse events stratified according to type of side effect and severity (any grade or severe adverse event).

Event	Any grade N (%)	Grade≥3 N (%)
Fatigue (n=240)	146 (61%)	4 (2%)
Nausea (n=224)	60 (27%)	0
Anaemia (n=937)	634 (68%)	100 (11%)
Leukopenia (n=937)	335 (36%)	40 (4%)
Thrombocytopenia (n=937)	374 (40%)	52 (6%)
Renal function impairment (n=793)	334 (42%)	33 (4%)
Xerostomia (n=477)	365 (77%)	7 (2%)
